# Reversal of functional disorders by aspiration, expiration, and cough reflexes and their voluntary counterparts

**DOI:** 10.3389/fphys.2012.00467

**Published:** 2012-12-14

**Authors:** Zoltan Tomori, Viliam Donic, Roman Benacka, Sona Gresova, Igor Peregrim, Martin Kundrik, Maria Pallayova, Jan Jakus

**Affiliations:** ^1^Department of Human Physiology, Faculty of Medicine, University of PJ SafarikKosice, Slovakia; ^2^Department of Pathophysiology, Faculty of Medicine, University of PJ SafarikKosice, Slovakia; ^3^Department of Medical Biophysics, Jessenius Faculty of Medicine in Martin, Comenius University in BratislavaSlovakia

**Keywords:** aspiration reflex, breathing maneuvers, expiration reflex, functional disorders, urge-to-cough

## Abstract

Agonal gasping provoked by asphyxia can save ~15% of mammals even from untreated ventricular fibrillation (VF), but it fails to revive infants with sudden infant death syndrome (SIDS). Our systematic study of airway reflexes in cats and other animals indicated that in addition to cough, there are two distinct airway reflexes that may contribute to auto-resuscitation. Gasp- and sniff-like spasmodic inspirations (SIs) can be elicited by nasopharyngeal stimulation, strongly activating the brainstem generator for inspiration, which is also involved in the control of gasping. This “aspiration reflex” (AspR) is characterized by SI without subsequent active expiration and can be elicited during agonal gasping, caused by brainstem trans-sections in cats. Stimulation of the larynx can activate the generator for expiration to evoke the expiration reflex (ExpR), manifesting with prompt expiration without preceding inspiration. Stimulation of the oropharynx and lower airways provokes the cough reflex (CR) which results from activating of both generators. The powerful potential of the AspR resembling auto-resuscitation by gasping can influence the control mechanisms of vital functions, mediating reversal of various functional disorders. The AspR in cats interrupted hypoxic apnea, laryngo- and bronchospasm, apneusis and even transient asphyxic coma, and can normalize various hypo- and hyper-functional disorders. Introduction of a nasogastric catheter evoked similar SIs in premature infants and interrupted hiccough attacks in adults. Coughing on demand can prevent anaphylactic shock and resuscitate the pertinent subject. Sniff representing nasal inspiratory pressure and maximal inspiratory and expiratory pressures (MIP and MEP) are voluntary counterparts of airway reflexes, and are useful for diagnosis and therapy of various cardio-respiratory and neuromuscular disorders.

## Introduction

**Figure 1:**
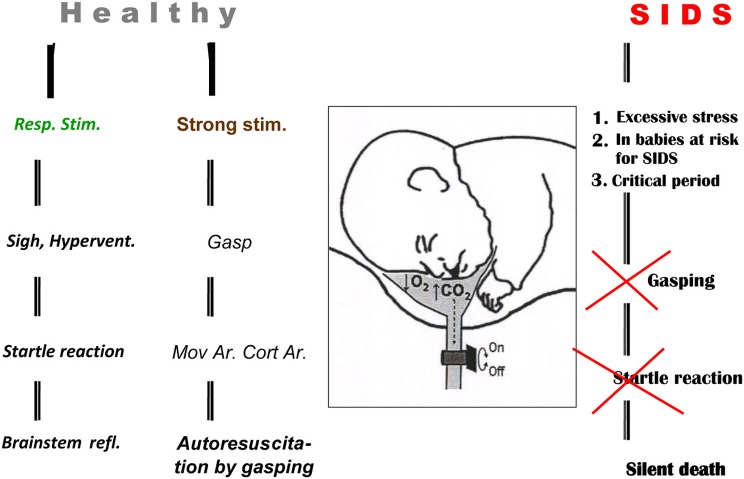
**Auto-resuscitation in healthy infant and its failure in SIDS baby.** Three different outcomes of respiratory reactions coupled with or without arousal (Ar) in infants are illustrated schematically: (1) normalization of vital functions, mediated by brainstem reflexes connected with sigh and startle reaction, provoked by milder respiratory stimuli without Cortical Arousal—Cort Ar. (2) Auto-resuscitation by gasping, coupled with Cort Ar, Movement Arousal—Mov Ar, where gasping is provoked by strong stimulation. (3) Death resulting from failure of vital functions caused by excessive stimulation or structural defects and insufficiency of the control mechanisms in babies at risk for SIDS. Reproduced with permission from a review by Tomori et al. (2010).

The gradual development of gasping in infants dying of sudden infant death syndrome (SIDS) has been detected by cardio-respiratory monitoring (Thach et al., 1989b; Wulbrand et al., 1995). Gasping also develops regularly in pigs and other animals during untreated experimental ventricular fibrillation (VF) after ~5 min (Xie et al., 2004). Such gasping can auto-resuscitate some of these animals (Thach et al., 1991; Thach, 2005). Clinical studies indicated that occlusion of a face-mask outlet at the end of inspiration for several seconds in infants during sleep, after transient inhibition gradually enhanced diaphragmal activity and caused a negative pressure in the mask. After development of hypoxia and hypercapnia *a sigh* occurred regularly. The augmented part of sigh was always accompanied with a startle reaction, manifesting with activation of the nuchal and limb muscles, which caused reopening of upper airways (UAs) in both REM and non-REM sleep. The startle reaction was usually accompanied with a micro-arousal lasting <3 s, the intensity of which correlated with a simultaneous heart rate acceleration. These results indicate that opening of the UAs by a sigh and startle can be realized by brainstem reflexes without cortical arousal (Wulbrand et al., 2008). Strong stimulation in healthy infants evokes a gasp, often resulting in *auto-resuscitation by gasping* (McNamara et al., 2002). Similar occlusive events occurring in babies at risk for SIDS during a critical period of development (mostly in 1st year) does not evoke a sigh, startle, or gasp, but results in silent death, as illustrated schematically (Figure 1).

Monitoring of infants during sleep indicated that occlusion of the face-mask outlet represents a strong stimulus, causing hypoxemia and hypercapnia. This was accompanied by sighs and gasps, often resulting in auto-resuscitation by gasping. Therefore, the explanation of the mechanisms of the auto-resuscitation by gasping appeared to be extremely important from both theoretical and practical points of view. Negative pressure applied to UAs strongly increases the inspiratory drive during sleep. More negative pressure than −12 cm H_2_O in the UAs induce a “*pharyngeal dilatory reflex*” (Thach et al., 1989a). Similar gasp- and sniff-like spasmodic inspirations (SIs), the so-called *aspiration reflex (AspR)* can be regularly induced by mechanical contact, pressure pulses, and electrical stimulation of the nasopharynx (NPh) in cats (Tomori and Widdicombe, 1969; Tomori, 1979b; Tomori et al., 2000, 2010). AspR has a powerful revitalization potential manifesting with reversal of hypoxic apnoea, asphyxia, and even a comatose state (Tomori et al., 1991, 1995a, 2000). Therefore, our aims were: (1) To analyze the effects of AspR and other airway reflexes for reversal of functional disorders in animals, and (2) to discuss the existence and applicability of airway reflexes and their voluntary counterparts (sniffs + prompt expirations) in the diagnostics and therapy of several cardio-respiratory and neuromuscular disorders in patients.

## Reflex modifications of breathing

### Upper airway negative pressure reflex

The *upper airway negative pressure reflex* (UANPR) is present in both animals and humans. Its afferent pathways were studied using anesthesia of UAs (Horner et al., 1991a,b). The reflex can change the lumen of UAs by activation of dilatory muscles: m. genioglossus (GG) and m. tensor palatini. Negative pressure pulses (−10 cm H_2_O lasting for 250 ms) during early inspiration in healthy men can evoke a short-latency activation followed by a suppression of the GG EMG in wakefulness and sleep. The valvular function of UAs relate both to airway patency (measured by pharyngeal resistance) and anatomical airway narrowing/collapsibility (reflected by positive end-expiratory pressure). In addition to primary activation the UANPR also induces a secondary inhibition of the GG muscle activity (67.8% of baseline), with a latency of 71 ± 4 ms in 35% of healthy subjects (Eckert et al., 2007, 2010). The aim of such secondary inhibition is probably to prevent an aspiration of the secretion into the lungs, allowing UAs collapsibility and modification of the diaphragmal activity.

### Defensive airway reflexes

Breathing and other vital functions are strongly influenced by various airway reflexes, including mainly coughing, and two other distinct reflexes, that have been systematically analyzed over multiple decades. A multifunctional respiratory pattern generator undergoes reconfiguration to produce cough and other airway defensive behaviors. Behavior selective neurons may be responsible for regulating the function of core pattern generating network to allow it to mediate multiple tasks, including amplification of the motor drive. Such task may be rhythmic as breathing and swallowing or highly patterned reactions such as cough, expiration, and AspRs (Bolser et al., 2006). Pre-Bötzinger complex (preBötzC) is necessary and sufficient for generating inspiratory rhythm. This produces normal respiratory activity (eupnea) and sighs under normoxic conditions, but gasping during hypoxia after a reconfiguration process. The reconfiguration involves changes in synaptic and intrinsic properties of neurons that can be mediated by several neuromodulators, including 5HT and substance P (SP). Neuromdulation provided mainly by glial cells are important for rhythmogenesis of preBötzC. Continuous neuromodulation tunes the excitability of the preBötzC to respond to different demands and also determines the weight of specific neuronal types or specific synaptic interactions with generation of network dynamics (Pena-Ortega, 2012). The extremely high excitability of inspiratory generator can explain the constant elicitability of strong gasp-like AspR by nasopharyngeal stimulation. It is a very important observation that each mechanical contact stimulation of the NPh during agonal stage in cats evokes even stronger gasp-like AspR than the spontaneous agonal gasp (Tomori, 1979b).

**Figure 2:**
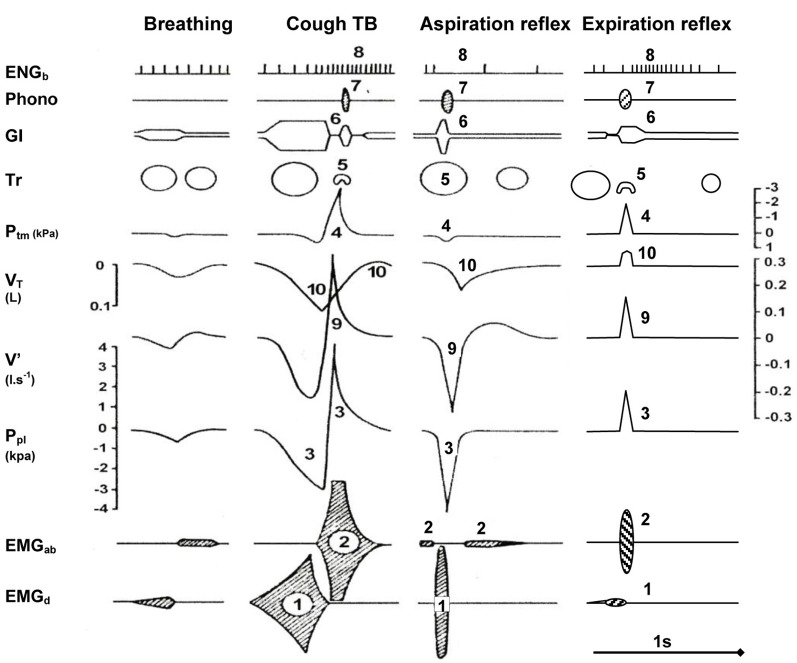
**Main components of selected airway reflexes compared to quiet breathing.** Schematic illustration of 10 main components (indicated by numbers) during one cycle of AspR and ExpR, compared with the cough reflex and quiet breathing in the anesthetized cat. Abbreviations: (1) EMG_d_, diaphragmal electromyogram; (2) EMG_ab_, abdominal electromyogram; (3) P_pl_, pleural pressure; (4) P_tm_, transmural pressure; (5) Tr, tracheal lumen; (6) Gl, glottal lumen; (7) Phono-acoustic signal of breathing; (8) ENG_b_, electroneurogram of a bronchoconstrictor fiber activity; (9) V', airflow; (10) V_T_, tidal volume. The axis for flow is on the right side of the figure but the flow label is on the left. Reproduced with permission from a review by Tomori et al. (2010).

Figure 2 schematically illustrates the 10 main components of the three most important defensive airway reflexes compared to quiet breathing (Tomori et al., 2010). *The cough reflex (CR)*, held as the “watchdog of the lungs” is induced mostly by stimulation of rapidly adapting receptors (RARs) from the tracheal-bronchial region. It consists of three phases and manifests with strong inspiratory and expiratory efforts (Korpas and Tomori, 1979; Jakus et al., 2004b; Widdicombe, 2006, 2011). During the initial deep inspiration (DI), the gradually increasing lung volume stimulates the slowly adapting receptors (SARs) of airways and *via* the Hering-Breuer inflation reflex (HBIR) induces the expiratory period, starting with the compressive phase. After glottal closure and rapid compression of the enlarged lung volume via the so-called *Hering-Breuer expirium facilitating reflex (HBEFR)* provokes strong expiratory effort (Marek et al., 2008), manifesting during the expulsive phase of CR. In addition to the HBIR and HBEFR, the proprioceptive reflexes of the respiratory muscles and stimulation of RARs, SARs, A_δ_, and C-fibers in the airways and lungs in non-paralyzed subjects, can also contribute to the modification of cough efforts (Korpas and Tomori, 1979; Widdicombe, 2006, 2011; Widdicombe et al., 2011a,b). Studies in guinea pigs suggest that a previously unrecognized subtype of airways afferent nerve—cough receptors—play the primary role in regulating this defensive reflex. These putative cough receptors are myelinated and do not synthesize and express neuropeptides under normal conditions (Canning and Mazzone, 2005). Experiments in rabbits and dogs suggest a significant role also for slowly adapting airway receptors in CR (Sant'Ambrogio et al., 1984).

Mechanical stimulation of the larynx in animals and humans evokes the *expiration reflex* (ExpR), characterized by prompt expiratory effort without preceding inspiration (Korpas, 1972, 1979; Tatar et al., 2008). When provoked during a spontaneous inspiration, ExpR, contrary to CR immediately interrupts the present inspiration, and evokes laryngo-constriction to prevent aspiration of the irritants into the lower airways and the lungs and expels them promptly by strong expiratory effort (Tomori et al., 2010; Widdicombe et al., 2011a). ExpR, similarly to CR is accompanied by bronchoconstriction in addition to laryngeal closure. On the contrary, AspR is accompanied by a dilation of the upper and lower airways (Figure 2).

Mechanical, electrical, and other methods of stimulation of the NPh in cats and most mammals regularly evoke *a sniff- and gasp-like AspR* (Tomori, 1965, 1979b; Donic et al., 1992). It manifests as a solitary SI that lasts only for 150–230 ms in the diaphragmal EMG and airflow records and is usually not followed by an immediate active expiration (Benacka and Tomori, 1995; Tomori et al., 1995b, 1998). The tendency to SI decreases gradually from the beginning of the inspiratory phase of breathing (Marek et al., 2008), probably reflecting the respiratory drive. Similar SIs were observed after induction of a nasogastric catheter for feeding also in premature infants (Javorka et al., 1980). In paralyzed cats, stimulation of the “irritant” RARs of the NPh by mechanical contact or pressure pulses evokes a very strong activity in glossopharyngeal afferents. The high frequency of impulses (mean 197/s, range 56–330), (Nail et al., 1969) strongly activates many inspiratory neurons in the brainstem (Jakus et al., 2004a,b), probably also including inspiratory neurons in the preBötzC, described recently as “*the generator for inspiration*” in rats (Janczewski and Feldman, 2006a,b). AspR and ExpR provide very strong separate reflex activations of the two distinct brainstem generators for inspiration and expiration with simultaneous reciprocal inhibition. Therefore, they provide a unique methodological basis for their practical applications in animal and clinical studies.

#### Specific characteristics and mechanisms of AspR

In a recent review of reflexes from the upper respiratory tract (in *Comprehensive Physiology*; Widdicombe, 2011) Widdicombe stated that the UA reflexes were extensively analyzed in a monograph by Korpas and Tomori (1979). He indicated that this book was the first work to study the AspR comprehensively, completing two basic papers (Tomori, 1965; Tomori and Widdicombe, 1969) in addition to cough and ExpRs. This reaction was later described as *the sniff reflex* (Batsel and Lines, 1973), but the distinction between sniffing induced by odors inhaled into the nose and AspR from the NPh is not very clear. This reaction was analyzed from 1994 also by Japanese researchers as *a hiccup-like reflex* (as discussed later). In a recent review, Widdicombe (2011) stated that AspR in cats is exceptionally resistant against anesthesia, hypothermia, asphyxia, and could be important in reanimation. This exceptional resistance is based on the fact that AspR can be evoked in anesthetized cats even after total brainstem transection 5 mm above the obex in the agonal state after elimination of the ExpR and CR and replacement of breathing by gasping respiration (Jakus et al., 1987, 2004a,b). Reflex recruitment of medullary gasping mechanisms can be induced by pharyngeal stimulation in cats during eupnea (Fung et al., 1994; Tomori et al., 1995a, 2010).

**Figure 3:**
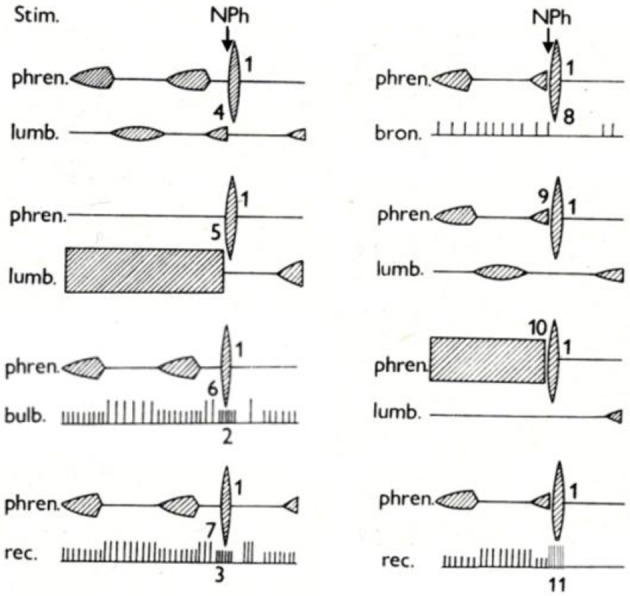
**Specific activity in various neural structures evoked by NPh stimulation in cats.** Schematic illustration of changes evoked in activity of motoneurones of the phrenic nerve (phren.), lumbar nerve (lumb.), and recurrent laryngeal nerve (rec.), of bulbar inspiratory and expiratory neurons (bulb.) and of bronchoconstrictor fiber of vagus (bron.) by mechanical stimulation (Stim.) of nasopharynx (NPh) in anesthetized cats. The numbers indicate development of specific activity in the phrenic nerve (1), in inspiratory neurons of medulla oblongata (2) and in inspiratory neurons of recurrent laryngeal nerve (3) during expiration. Inhibition of spontaneous (4) and tonic (5) expiratory activity in the lumbar nerve, bulbar expiratory neurons (6), and recurrent laryngeal nerve (7) during expiration. Inhibition of bronchomotor fibers of vagus (8). Replacement of spontaneous (9) and tonic (10) inspiratory activity in the phrenic nerve and recurrent laryngeal nerve (11) by specific activity during inspiration. Adapted from Tomori et al. (1976).

*The specific character and the complex effects of AspR* are schematically illustrated in Figure 3. It indicates that AspR can be evoked in any phase of the respiratory cycle in a cat. It replaces the spontaneous inspiration or expiration by a rapid and strong spasmodic inspiratory effort and UA dilation, caused by strong activities in the phrenic and superior laryngeal nerves, and bulbar inspiratory neurons. It can also replace strong tonic expiratory activities in the lumbar and laryngeal recurrent nerves. Also, bulbar expiratory and inspiratory activities can be suppressed and replaced, and the bronchoconstrictor nerve activity can be inhibited by the powerful activity of AspR. These effects suggest practical applicability of AspR for inhibition of unwanted effects of coughing, asthmatic attacks, spastic events, etc.

**Figure 4:**
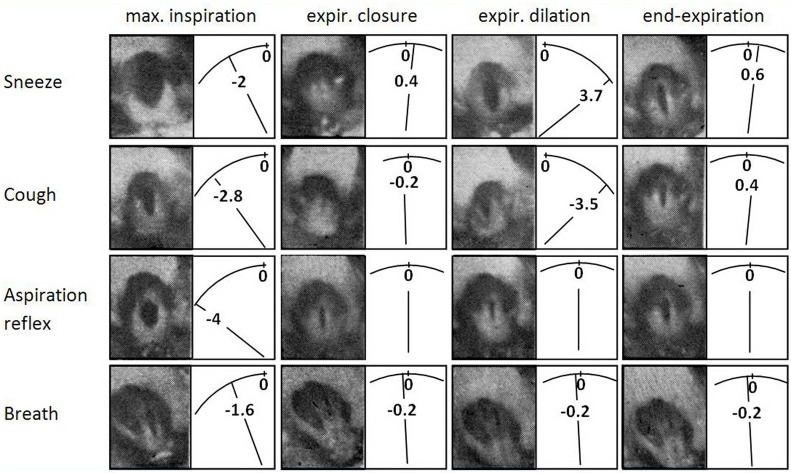
**Cine-photographs of glottis in sneeze, cough, AspR, and control breath in a cat.** Cine-photographs of glottis in one inspiratory—expiratory cycle of the sneeze, cough from tracheo-bronchial region, aspiration reflex, and control breath in anesthetized cat. Phases illustrated: maximum inspiration, early expiratory glottal closure or narrowing, successive expiratory glottal dilation, and end of expiratory phase. Instantaneous pleural pressure (P_pl_) values were recorded in parallel, are illustrated diagrammatically and expressed in kPa to the right of each picture. The figure was reproduced with permission from a chapter “Tomori: The Physiology of the cough reflex, 1979a,” in a monograph “Korpas and Tomori: *Cough and Other Respiratory Reflexes*,” published by Karger in Basel.

**Figure 5:**
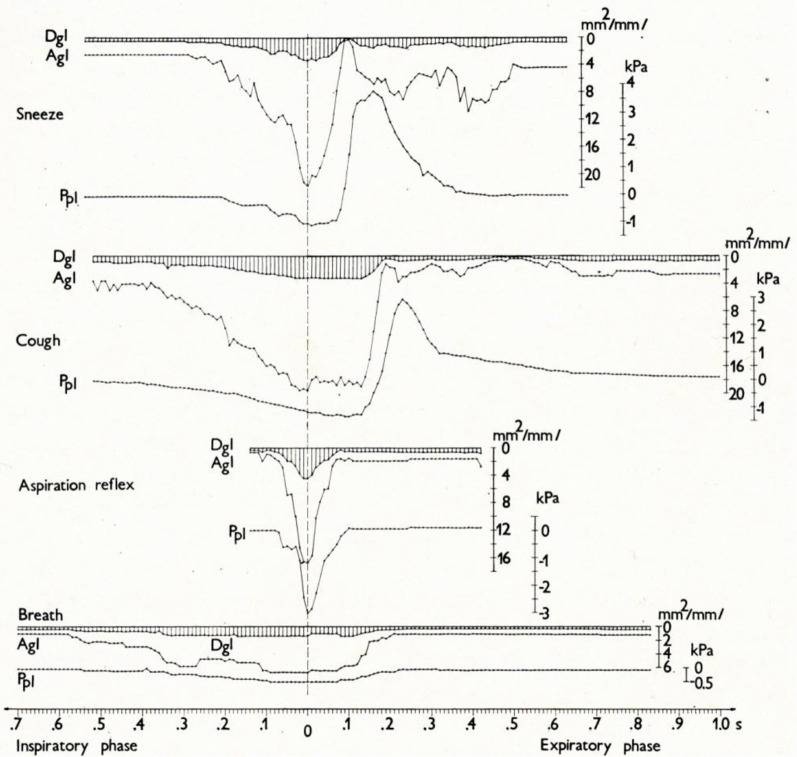
**Changes in glottal diameter and area, and pleural pressure in the sneeze, cough, AspR, and control breath in cats.** Dynamics of synchronous changes in glottal diameter (Dgl) and area (Agl) as well as in instantaneous pleural pressure (Ppl) during one inspiratory-expiratory cycle of the sneeze, cough, aspiration reflex, and control breath. Mean values for anesthetized cats are indicated schematically. The transition from the inspiratory to the expiratory phase after the moment of maximum glottal dilation is indicated by the vertical broken line. The figure was reproduced with permission from a chapter “Tomori: The Physiology of the cough reflex, 1979a,” in a monograph “Korpas and Tomori: *Cough and Other Respiratory Reflexes*,” published by Karger in Basel.

*Cine-photography of the glottis* and parallel recording of *instantaneous pleural pressure* (Ppl) of one cycle of typical AspR in a cat are compared with other airway reflexes in Figures 4, 5. The data indicate that in AspR, the rapidly starting maximal inspiratory Ppl is more negative and the trans-glottal diameter and glottal area are similar, than during the inspiratory phase of CR. During the second part of the cycle, the trans-glottal diameter and glottal area in AspR decreased in parallel with the decrementing negative Ppl, causing some acoustic signal. However, contrary to the cough and sneeze, there is no total closure of glottis or active expiration, similar to quiet expiration. In spontaneous gasp and AspR provoked by NPh stimulation, there is a tendency for maximal contraction of the inspiratory muscles of *all or nothing* type with a simultaneous inhibition of expiratory muscle activity and rapid glottal opening. These activities tend to supply oxygen to the lungs and also enhance the venous return of blood to the heart, due to a strong negative intrathoracic pressure. They support the perfusion of the myocardium and brain. Therefore, they can prevent an imminent loss of consciousness and promote the auto-resuscitation (Tomori, 1979b; Tomori et al., 2010). A transient activation of the laryngeal adductor muscles prior to a strong glottal dilation and powerful diaphragmal activity, tend to stiffen the UA wall to prevent collapse and total closure of glottis (Tomori, 1979b; Poliacek et al., 2003).

#### The hiccough-like reflex

A reaction very similar to the sniff- and gasp-like AspR (illustrated in Figures 4, 5) was later analyzed by Japanese authors in cats and described independently as a *hiccough-like reflex*. This reaction was elicited from the epipharyngeal area (Oshima et al., 1994). It also has a glosso-pharyngeal afferent pathway (Kondo et al., 2003) and involves similar brainstem inspiratory neurons (Arita et al., 1994). In addition it has also a very powerful inspiratory activity (Oshima et al., 1998) as the AspR analyzed by us. In this hiccough-like reflex, there was a glottal closure, presumably caused by a secondary reflex suppression in genioglossal muscle activity observed by Eckert et al. (2010), as a response to the UANPR. The AspR resembles a gasp and sniff, rather than hiccough, as shown by the difference in the acoustic signal and the lack of total expiratory closure of glottis, documented in Figures 4, 5, adapted from our monograph (Korpas and Tomori, 1979).

Hiccough caused very serious diagnostic and therapeutic problems, indicated in ~2000 contributions according to a search on terms “hiccough,“ “hiccup-like reflex,” and “singultus” using PubMed. Hiccough can be evoked in 40% of healthy participants by rapid distension of the upper part of esophagus with a balloon inflated with 40 ml air for 1 min, representing a pressure of 64 mmHg (Fass et al., 1997). The functional role of hiccough is not clear (Kahrilas and Shi, 1997). The distressing hiccough attacks in patients, however, can be interrupted by introduction of a nasogastric catheter to the cervical region (Salem et al., 1967), provoking probably a gasp-like reaction. An even more practical and truly working method for hiccough interruption is swallowing of 400 ml cold water, combined with a tight pressure on tragi of ears inward to completely seal the external acoustic meati while the patients simultaneously drink all the water through a straw. By the end of this maneuver, the hiccoughs should have resolved (Goldstein, 1999).

#### Gasp-like reactions to stimulation of highly sensitive superficial points

Similar sigh- and gasp-like SIs resembling AspR, being evoked by stimulation of the NPh, can be provoked by stimulation of several highly sensitive superficial locations as well. Three devices and methods are now being used by us for reversal of several functional disorders, not caused by irreversible structural alterations or chronic pathological processes. A series of such SIs are accompanied by activation of the sympathetic nervous system, resulting in tachycardia, vasoconstriction, and hypertensive reaction, but also with signs of Micro-Arousal characterized by 1.5–3 s alpha rhythm in EEG and brainstem acoustic-evoked potentials (BAEP). Such SIs evoked by stimulation of nasal filter can repeatedly interrupt severe hypoxic apnea and even a developing comatose state in anesthetized cats (Benacka and Tomori, 1999).

### Characterization of various types of deep and spasmodic inspirations

There are many types of inspiration, where the activity of respiratory muscles, valvular function of the glottis, the airflow rate, and the inspired volume differ but are pertinent to the behavioral task. DI is usually the first phase of *CR*, substantially increasing the successive expiratory effort (Tomori and Widdicombe, 1969; Tomori, 1979a; Marek et al., 2008) as discussed earlier. *A sniff* promoting olfaction requires a short-lasting rapid inspiration with a strong but transient glottal opening and a moderately augmented tidal volume. This is necessary for the transport of odorants with airflow mainly to the olfactory receptors in the upper part of nasal cavity, but not to the lungs. At small concentrations of the odors, the inspired volume increases automatically to compensate for the low intensity of olfactory stimulation by an increase in volume. *In hiccough*, there is a late-inspiratory sudden glottal narrowing, accompanied by a typical acoustic phenomenon. This is caused by movement of inspired air through the decreasing glottal area, preventing larger lung inflation, in spite of the strong spasmodic twitch of inspiratory muscles (Newsom Davis, 1970).

**Figure 6:**
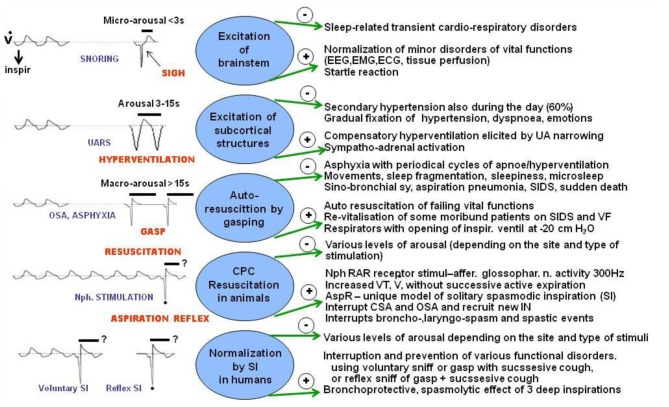
**Effects of various respiratory reactions coupled with different degrees of arousal compared with AspR and its counterparts.** Schematic illustration of negative (−) and positive (+) effects of various respiratory efforts coupled with different levels of arousal, gasping auto-resuscitation, and revitalization by AspR and spasmodic inspirations. Some effects, caused by modification of breathing coupled with arousal reactions during sleep in patients mediated by variable neuronal substrates are compared with changes provoked by AspR elicited by mechanical stimulation of nasopharynx (NPh) in anesthetized cats and with presupposed effects of reflex and voluntary spasmodic inspirations (sniffs) in humans.

Schematic Figure 6 indicates various forms of respiratory efforts, connected with different degrees of arousal reactions, mediated by variable neural substrates. In the figure, the negative (−) and positive (+) effects of these arousal reactions in humans, are compared with AspR induced by nasopharyngeal stimulation in cats. Milder sleep-related breathing disorders, such as *snoring* and upper airway resistance syndrome (UARS), accompanied by Micro-Arousal or short-Arousals mainly tend to normalize the separate transient disorders of vital functions. *In sigh*, there is a second augmented part, representing a SI with a wide opening of the glottis. This allows lung inflation with distension of the atelectatic regions, which can persist. More severe degrees of sleep disordered breathing (SDB), often connected with asphyxia are accompanied by Macro-Arousal and causing *solitary gasps*. Gasps may normalize breathing, resulting in adaptation to hypoxia by preconditioning. Progressive hypoxia and asphyxia may result in auto-resuscitation by development of *periodic gasping*. However, the airway protective reflexes, mainly the cough, ExpR, and swallowing, as well as general defense mechanisms including muco-ciliary transport, antibacterial and antiviral defense, alveolar macrophages, may fail. Then the persisting gasps may contribute to development of a sino-bronchial syndrome or aspiration pneumonia (Addington et al., 2003, 2005; Widdicombe et al., 2011a). Also severe nocturnal cardiac arrhythmias may develop in patients with SDB and their occurrence correlates with intensity of sleep apnea in men (Szaboova et al., 2013). Even a sudden cardiac death may occur, particularly in elderly patients and during frequent respiratory infections.

*The AspR* induced by stimulation of NPh in cats activates many brainstem inspiratory neurons, and generates a gasping-like activity (Fung et al., 1994; Tomori et al., 1995a). Some of these neurons were recently specified as the “generator for inspiration,” in the medullary preBötzC of neonatal rats (Janczewski and Feldman, 2006a,b). AspR can influence also the central mechanisms of various vital functions through dense synaptic connections in rats (Tan et al., 2008, 2010), as well as in cats (Jakus et al., 2004b). Therefore, AspR could interrupt various cardio-respiratory and neurobehavioral disorders of functional character (Tomori et al., 2010), via influencing 14 of 35 nuclei identified in the brainstem of cats (Jakus et al., 2004a).

### Revitalization effects of deep and spasmodic inspirations and prompt expirations

**Figure 7:**
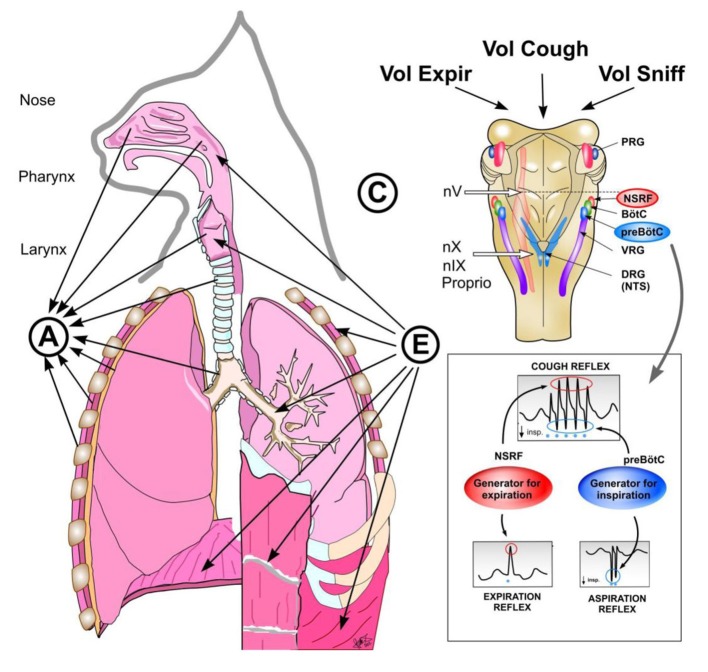
**Reflex arch and mechanisms of AspR, ExpR, and CR and their voluntary counterparts.** Schematic illustration of reflex arch and mechanisms of AspR, ExpR, and CR and their voluntary counterparts. A—afferents via trigeminal nerve from the upper part of nasal cavity for sniffing, via n. glosso-pharyngicus from the nasopharynx for AspR, via n. laryngicus sup. from the larynx for ExpR and via n. vagus from tracheal-bronchial region and proprioceptive afferents for CR. E—efferents to inspiratory muscles (diaphragm and external intercostals) and to expiratory muscles (abdominal and internal intercostals) for ExpR and CR. C—central structures: Generator for inspiration in preBötzinger complex for AspR, generator for expiration in NSRF for ExpR and both generators for CR. Breathing maneuvers allow voluntary performance of all three reflexes (voluntary sniffs, prompt expiration, and sniff followed by strong expiration), which have perspective clinical applications.

The three airway reflexes very strongly activate the two brainstem generators (Figure 7) and can directly *modify (facilitate or inhibit) the* “*central control mechanisms*” *of various vital functions* (Tomori et al., 2010). They are very effectively mediated by dense synaptic connections (Tan et al., 2010) and various mediators. Such “overdrive activation of the inspiratory generator” caused by NPh stimulation provokes AspR, which might provide a unique chance to reset the control mechanisms, primarily of three vital functions (breathing, cardio-vascular, and neuromuscular activities). This might promote normalization of hypo- and hyper-functional disorders, if not hindered by the presence of severe or fixed changes (e.g., acute stroke or recent myocardial infarction), both in animal experiments and probably also in human studies. *AspR* and *ExpR* reversed several life-threatening disorders of functional character, manifesting as apnea termination, and resuscitation in cats (Tomori et al., 2000). Power spectral analysis of the phrenic and the hypoglossal nerve activity in paralyzed cats indicated very similar peaks during both the hypoxic gasping and AspR, evoked by NPh stimulation (Fung et al., 1994; Tomori et al., 1995a,b), resembling *auto-resuscitation by gasping*. Therefore, the powerful AspR might influence positively the imbalance between the excitatory and inhibitory cardio-respiratory reflexes responsible for survival or death in the patho-mechanism of SIDS, according to the hypothesis of Leiter and Böhm (2007).

Sniff-like AspR, ExpR, and CR, as well as the UA negative pressure reflex, and particularly their voluntary counterparts, *the sniff* plus *forced expiration*, have complex central control mechanisms. Their interaction and support by behavioral and pharmaceutical methods might have a great practical importance. AspR often has a multi-phasic character, manifesting with several repetitive SIs, particularly in cases of hyperreactivity or frequent intensive natural or experimental stimulation. It resembles the multiple “double” and “triple” gasps, observed frequently in moribund infants with SIDS (Sridhar et al., 2003; Wulbrand et al., 2008). Similarly, 2–3 successive active expirations without interposed inspirations may manifest as separate ExpRs in a cough attack, occurring occasionally in cats with experimental bronchitis (Korpas, 1979). Many functional disorders might be modified by SIs, induced by nasopharyngeal stimulation or by various other methods of SIs provocation. Different patho-physiological conditions could also be normalized, by using AspR and ExpR directly, or through the dense synaptic connections of the brainstem “inspiratory generator,” and “expiratory generator” or by a variety of neuromodulators. However, breathing is a kind of voluntary somato-motor activity that depends on the integration of a chemosensory drive, arising from the central and peripheral chemoreceptors with dynamic feedback from airway afferents and with the respiratory rhythm generator (McCrimmon and Alheid, 2007). In this complex control mechanism, the airway reflexes and particularly the AspR may have an important restorative and life-saving contribution and their applicability and mechanisms have to be tested both in animal experiments and clinical studies.

Stimulation of the larynx both in cats and humans strongly activates the higher located generator for expiration causing laryngo-constriction and prompt expiratory effort without preceding inspiration. This prevents intrusion of secretions and irritant materials into the lungs and supports their expulsion. Stimulation of the tracheo-bronchial mucous membrane activates both the inspiratory and expiratory generators provoking DI followed by a powerful expiratory effort for prevention of aspiration into the lungs. It also provides venous return to the heart and supports brain perfusion preventing and terminating various collapsible states. Therefore, *AspR as a model of SIs* alone or combined with *forced expiration (ExpR)* or *prompt voluntary cough effort* (without preceding inspiration) can serve to reverse many dysfunctional states. These “on demand” provocations could be very useful for the study of cardio pulmonary cerebral resuscitation (CPCR) in model experiments with dysrhythmias, including VF, particularly in pigs, cats, mini pigs, and in rats. Already spontaneous gasps restored the cerebral blood flow (CBF) to 59% of the control values. There was a very significant correlation of CBF with decreases in intrathoracic pressure during the inspiratory phase of gasps and with the increases of aortic pressure during the expiratory phase of gasps. Spontaneous gasps producing significant increases in the CBF during untreated cardiac arrest, confirmed the beneficial resuscitative effect of gasping during the cardiac arrest (Ristagno et al., 2007). Similarly, reversal of many other patho-physiological conditions, indicated in Figure 6, could be tested by using AspR and ExpR or their voluntary counterparts.

In ExpR there is a very positive correlation between the lung inflation volume and pressure, provoked during the expiratory period of the respiratory cycle (Poliacek et al., 2008). This relation can be explained by HBEFR, which is very strong at the beginning of the expiratory period, reflecting the momentary large lung volume (Marek et al., 2008). In addition to strong gasp-like inspirations provoked by NPh stimulation and mediated by brainstem central control mechanisms participating in gasping, the AspR is also characterized by reciprocal inhibition of expiratory activity. Experiments in paralyzed cats, however, allow evaluation of persisting *reflex effects of the* “*fictive AspR*” (Shiba et al., 1999). Vegetative reflex tachycardia and vasoconstriction evoked by fictive AspR may at least partially support auto-resuscitation after myo-neural blockade supporting venous return to the heart and brain.

In a similar manner, 2–3 *voluntary rapid and strong sniffs*, each followed by a prompt forceful expiratory effort, may prevent an imminent loss of consciousness. They may even resuscitate the subject, resembling auto-resuscitation by gasping (Figure 6). In addition to cardiovascular applications aimed to treat severe morbid cases and prevent a sudden cardiac death, a combination of AspR, supporting venous return and ExpR, or a prompt cough effort, increasing cerebral perfusion, provide a simple but unique model tested by us now both in animal experiments and human studies.

## Cortical and sub-cortical mechanisms of airway reflexes and breathing maneuvers

### Diagnostic and therapeutic applications of airway reflexes and respiratory muscle strength measurement

The strength of the protective and expulsive efficacy of airway reflexes is frequently measured by spirometry [forced expiratory volume/s (FEV_1_)], using inhalation of metacholine or citric acid mostly in asthma and chronic obstructive pulmonary disease (COPD). AspR has been described in cats (Tomori and Widdicombe, 1969) and its bronchodilatory effects were reproduced in adults with bronchial asthma (Legath et al., 2013). The CR hyper-sensitivity to capsaicin inhalation in asthmatic children was also decreased by proceeding voluntary sniffs (Pecova et al., 2013). *Determination of inspiratory and/or expiratory muscle strength* (EMS) also has a great diagnostic and therapeutic importance, mainly in severe respiratory and neuromuscular diseases. Sniff nasal inspiratory pressure (SNIP) represents a more simple method than the measurement of esophageal or oral maximal inspiratory and expiratory pressures (MIP and MEP) in patients with motor neuron disease (Choudri et al., 2000). The assessment of SNIP was successfully used as a simple alternative to MIP (Prigent et al., 2004). In COPD, in addition to the more complicated measurement of MIP, the traditional sniff maneuver and its provocation by cervical magnetic stimulation were used for determination of maximal diaphragm strength (Martinez-Lorens et al., 2006). The *sniff maneuver*, documented by measurement of SNIP, predicted and even prolonged the survival of patients with amyotrophic lateral sclerosis (Morgan et al., 2005) and facilitated weaning from mechanical ventilation (Moodie et al., 2011). Acute cardio-respiratory effects of EMS application were analyzed recently by Laciuga et al. (2012).

Experiments in cats indicated that mechanical stimulation of trachea evoked a cough reaction, which in 2/3 of cases started with ExpR-like response, which was weaker, and has a lower frequency than the typical ExpR from the larynx (Poliacek et al., 2008). The measurement of EMS could be useful for quantification and differentiation of the efficacy of ExpR evoked by stimuli from the larynx comprising laryngo-constriction and the expiration-like reflex and CR from the tracheo-bronchial area. There may be a difference in reaction to airway sensory stimuli evoking ExpR with the immediate inhibitory effect on inspiration and the strong CR, starting with a DI, which might contribute to the aspiration of infected materials into the lungs. Both the lower frequency and intensity of ExpR-like response from the tracheo-bronchial area depends on the momentary excitability of the brainstem generators for inspiration and expiration, respectively, which decreases during the pertinent inspiratory and expiratory phase, reflecting the instantaneous lung volume. However, the prevalence and intensity of activity of the pertinent generator will depend on the character and grade of the respiratory drive (McCrimmon and Alheid, 2007).

### Airway reflexes and breathing maneuvers in pathological conditions

In addition to characterization of the mechanisms and effects of different respiratory reflexes, their modifications, and practical applications in various pathological conditions are even more important. Particularly the transfer of results, proved in experiments to clinical practice is very prospective. Such situation was analyzed during a workshop: “Tuning the cough center,” which may manifest by stimulation, inhibition, sensitization, desensitization, or modification of the pattern of respiratory muscular contraction. For example, the main pulmonary afferent inputs, related to tuning the cough center are the cough receptors themselves, RARs, SARs, bronchial and pulmonary C fibers, and Aδ fibers. Therefore, the “cough reflex” is not a stereotyped response with single pattern, but is a range of responses, which can be modulated by a large variety of neural inputs substantially contributing to respiratory plasticity (Widdicombe et al., 2011a).

The loaded respiratory muscles have an exceptional potential to increase their activity (Ross et al., 2007), which can contribute to the prevention of respiratory insufficiency during the development of cardio-respiratory failure. Recent studies indicate that reduced respiratory neuronal activity elicits a unique form of plasticity in respiratory motor control, termed as *inactivity-induced phrenic motor facilitation*. It can be triggered by inhibition of breathing lasting even for 30 min, resulting from severe hypocapnia provoked by preceding hyperventilation, but also by very deep anesthesia, reflex vagal inhibition, and severe hypoxia. This may result in exaggerated activation of the brainstem generator for inspiration causing a facilitation of the inspiratory muscle activity. This situation can manifest with development of crossed phrenic phenomenon after spinal cord hemi-section or injury (Goshgarian, 2003), or as light-induced rescue of breathing after spinal cord injury (Alilain et al., 2008). Various forms of respiratory plasticity induced by intermittent hypoxia, which interact and ultimately impact on breathing stability, were investigated by Mateika and Narwani (2009). The mechanism of the reduced respiratory neural activity which elicits phrenic motor facilitation is analyzed more in detail by Mahamed et al. (2011).

It would have a great practical importance to test, if AspR and other SIs, or sniffing as their voluntary counterpart, could restore disrupted breathing, caused by various types of asphyxia. It is probable that the inactivity-induced facilitation of phrenic motoneurones has similar mechanisms as provocation of gasping respiration. The very resistant AspR persists even during the stage of pre-mortal gasping at least in cats (Jakus et al., 1987; Tomori et al., 2010). This allows a presumption that repeated AspRs and similar reflex SIs might provoke facilitation after a shorter period of breathing inactivity, rather than after a longer-lasting development of spontaneous gasping. Voluntary sniffs as counterparts of AspR might have similar restorative and resuscitative effects, particularly when applied at the beginning of spontaneous inspiration if present, when the inspiratory drive is maximal (Marek et al., 2008). Therefore, testing of the potential of AspR, ExpR, CR, and their voluntary counterparts for normalization and renewal of several functional disorders and even auto-resuscitation is an important goal.

The *CR* with the *ExpR* and *swallowing* provides the basic respiratory defense mechanisms preventing development of dangerous aspiration pneumonia, and these behaviors frequently fail particularly in elderly patients and in chronic respiratory infections. In elderly people the life-threatening pneumonia is very high, particularly in patients with multiple or bilateral cerebral infarction. The mechanism of aspiration pneumonia can be explained in part by the SP theory, supported by 7-times lower level of SP in sputum of such patients compared to healthy adults (Nakagawa et al., 1995). Experiments indicated that repeated applications of a dopamine D1 receptor antagonist inhibits swallowing reflex in guinea pigs (Jia et al., 1998). The decreased dopamine production may reduce the expression of SP at the glossopharyngeal nerve and at the cervical parasympathetic ganglion of the sensory branch of that nerve, representing the afferent neural pathways in cough (Karlsson et al., 1998). The depression of SP in insular cortex is thought to be associated with impairment of swallowing and consecutively of the CR and probably also the ExpR, which is the main reflex that should prevent aspiration (Ebihara and Ebihara, 2011). Their novel strategy for preventing aspiration pneumonia is based on a support of swallowing, coughing, and ExpR via insular cortex stimulation by thermal and olfactory stimuli in the food and by frequent oral care.

### The urge-to-cough model

The CR consists of three motor stages which may be modified by pathological conditions and three sensory stages which can be influenced voluntarily. In conscious adults, the urge-to-cough model allows prevention and treatment of unwanted aspirations, connected with impairment of swallowing and coughing as well as with DIs, which may contribute to development of dangerous aspiration pneumonia and other complications in patients at risk (Davenport, 2008). The onset of cough caused by hyperreactivity can be inhibited by behavioral methods in addition to pharmaceutical treatment. In gastro-esophageal reflux or intrusion of foreign substances into the pharynx with or without aspiration to the larynx and lower airways, reflex or voluntary swallowing and a prompt expiratory effort is required for expulsion of irritant substances. Stimulation of the oropharyngeal mucosa by air puff pressure pulses of −25 cm H_2_O in healthy adults-induced urge-to-cough, which only in 81% was followed by a CR. This indicates that the threshold for motor effect is higher, than for the sensory reaction (Wheeler-Hegland et al., 2011). In addition, healthy adults can modify voluntarily the CR motor output induced by inhalation of 200 μmol capsaicin (Hegland et al., 2012). A voluntary suppression of urge-to-cough may prevent unwanted coughing with DI and a subsequent risk for development of aspiration pneumonia. The onset of the cough expiratory effort may be also postponed and preceded by a breath-holding and swallowing of the bolus to the esophagus (Vertigan and Gibson, 2011).

For therapy of neuromuscular and respiratory disorders and diseases both expiratory and inspiratory muscle strength training proved to be a very promising method. For example, EMST for improvement of hypofunctional CR in older people, particularly with Parkinson's disease (PD). Dysphagia is the main cause of aspiration pneumonia and death in PD. IMST was used to improve weaning from mechanical ventilation. The mechanism of disorders of defensive mechanisms may be explained by improved hyolaryngeal complex movement (Troche et al., 2010). Cough provides high expiratory airflows to aerosolize and remove material that cannot be adequately removed by ciliary action. Cough and ExpR are particularly important for clearing foreign particles from the airways in those with dysphagia who may be at risk for aspiration pneumonia. After 4 weeks of EMST there was a significant decrease in the compressive phase duration and expiratory phase rise time, resulting in a significant increase in cough volume acceleration. As well, there was a significant decrease in the swallowing penetration/aspiration score, indicating that EMST is associated with improvement of both coughing and swallowing (Pitts et al., 2009). Analysis of the cortical gating mechanism indicated that oropharyngeal mechanosensation is not gated like other somatosensory systems (Poliacek et al., 2009a,b; Wheeler-Hegland et al., 2011).

### Evaluation of various components of airway reflexes and their voluntary counterparts

The particular phases of AspR, ExpR, and CR could be visualized by a stereo camera and calcium sensitive dyes in *in vitro* brainstem preparation (Koshiya and Smith, 1999). Such three-dimensional visualization can support better understanding of their mechanisms. The motor pattern of various airway defensive behaviors have spatiotemporal regulation. In the case of CR there is a separate regulation of the amplitude and temporal features of cough motor pattern. The time of cough expiration is determined by the second non-active phase of cough expiration (Bolser et al., 2006).

Functional magnetic resonance imaging (fMRI) and other methods were already tested for evaluation of sniffing and coughing (Simonyan et al., 2007). In spite of the undefined distinction between sniffing induced by odorous stimulation and AspR from the NPh (Widdicombe, 2011), sniffs and prompt forced expirations as voluntary counterparts of AspR and ExpR can provide an environmental control. The patients may be able to use these airflow behaviors to control/activate computer controlled devices. Therefore, even the severely disabled patients can communicate using a computer and regulate the movement of their wheelchairs, as was proved by Plotkin et al. (2010). Such activity can be realized in paraplegics by persistent voluntary sniffs and ExpRs, acting *via* UA reflexes representing binary symbols, that enable control of devices assisting in everyday activities.

fMRI revealed a preparatory activity to movement in the pre-motor area of the brain without a successive motor effect in the vegetative state, which was observed in some patients after verbal instructions (Simonyan et al., 2007). Marked activity was observed in the pre-motor cortex in vegetative state also by Beckinschtein et al. (2011). The persisting AspR might allow differentiation of patients in a vegetative state by the presence of activity in the pre-motor area of brain in fMRI on the instruction to move the hand, from non-reacting patient with brain death. Cardiogenic oscillation and ventilatory auto-triggering observed by Arbour (2009), and other signs of persistent vitality, detected by Wijdicks et al. (2010), could also be tested using airway reflexes. Therefore, similar activities might also be observed by fMRI after provocation of AspR by mechanical means even in more severe disorders of consciousness without voluntary but persisting reflex signs. Since AspR persists even in the stage of pre-mortal gasping at least in cats (Jakus et al., 1987; Tomori et al., 2010), the procedure might allow exclusion of brain death in selected and indicated cases of moribund patients.

## Conclusion

In our long-term study of airway reflexes two distinct reflexes were described in cats in addition to systematic analysis of cough. The sniff- and gasp-like AspR evoked by nasopharyngeal stimulation resembles gasping, which resuscitated ~15% of moribund infants and animals according to reserves of their vital functions. AspR in cats was proved to reverse hypoxic apnoea, laryngospasm, bronchospasm, and even transient comatose states. AspR followed by ExpR or a prompt cough effort may provide sufficient venous return to the heart and thus support the cerebral perfusion, providing CPCR, resembling auto-resuscitation by gasping. AspR strongly activates the brainstem inspiratory generator and ExpR the expiratory one. They can reverse many hypo- and hyper-functional disorders by influencing their central control mechanisms. Esophageal MIP and MEP and a very simple SNIP are often measured in asthma, COPD, weaning from non-invasive ventilation and even in Amyotrophic lateral sclerosis. EMST proved to be a useful method for support of airway defense by improvement of the swallowing and cough for prevention of aspiration pneumonia, particularly in patients with PD. Sniffs and strong expirations held as voluntary counterparts of AspR and ExpR might help to explain and quantify their presupposed reflex effects in clinical studies of various respiratory, cardiovascular, and neuromuscular diseases. Such breathing maneuvers providing a binary symbol (sniffs and prompt expirations), might enable severely disabled patients to communicate using a computer and control their wheelchair movement. Several effects are currently tested in animal experiments and human studies for implementation of the useful potential of airway reflexes and their voluntary counterparts.

### Conflict of interest statement

Prof. Zoltan Tomori and Prof. Viliam Donic are consultants of Nasophlex, Slovakia from 2009 with no influence on the present paper. Three patent applications were submitted by Gerrit deVos and Zoltan Tomori with the help of Nasophlex, Slovakia for provocation of AspR or spasmodic inspirations: (1) by stimulation of the nasopharynx, (2) by stimulation of the nasal philter, and (3) by stimulating a superficial point of a human ear, which are now tested in animal and clinical studies. The other authors declare that the research was conducted in the absence of any commercial or financial relationships that could be construed as a potential conflict of interest.

## Abbreviations

5HT: 5 hydroxy tryptamine
AspR: aspiration reflex
BAEP: brainstem acoustic-evoked potentials
CBF: cerebral blood flow
CD: cardiac death
COPD: chronic obstructive pulmonary disease
CPCR: cardio pulmonary cerebral resuscitation
CR: cough reflex
CSA: central sleep apnoe
DI: deep inspiration
ECG: electrocardiogram
EEG: electroencephalogram
EMG: electromyogram
EMS: expiratory muscular strength
EMST: expiratory muscular strength training
ExpR: expiration reflex
FEV_1_: forced expiratory volume/s
fMRI: functional magnetic resonance imaging
HBEFR: Hering-Breuer expiration facilitating Reflex
HBIR: Hering-Breuer inflation reflex
GG: genioglossus
IMST: inspiratory muscular strength training
IN: inspiratory neurons
MEP: maximal expiratory pressure
MIP: maximal inspiratory pressure
NPh: nasopharynx
NSRF: nucleus sub retro facialis
OSA: obstructive sleep apnoe
PD: Parkinson's disease
Ppl: pleural pressure
preBötzC: preBötzinger complex
RARs: rapidly adapting receptors
SARs: slowly adapting receptors
SDB: sleep disordered breathing
SI: spasmodic inspiration
SIDS: sudden infant death syndrome
SNIP: sniff nasal inspiratory pressure
UAs: upper airways
UANPR: upper airway negative pressure reflex
UARS: upper airway resistance syndrome
V': airflow
VF: ventricular fibrillation
V_T_: tidal volume.
